# Molecular biology of Hodgkin lymphoma

**DOI:** 10.1038/s41375-021-01204-6

**Published:** 2021-03-08

**Authors:** Marc A. Weniger, Ralf Küppers

**Affiliations:** Medical Faculty, Institute of Cell Biology (Cancer Research), University of Duisburg-Essen, Essen, Germany

**Keywords:** Cancer genetics, Oncogenesis, Tumour immunology

## Abstract

Classical Hodgkin lymphoma (cHL) is unique among lymphoid malignancies in several key biological features. (i) The Hodgkin and Reed-Sternberg (HRS) tumor cells are rare among an extensive and complex microenvironment. (ii) They derive from B cells, but have largely lost the B-cell typical gene expression program. (iii) Their specific origin appears to be pre-apoptotic germinal center (GC) B cells. (iv) They consistently develop bi- or multinucleated Reed-Sternberg cells from mononuclear Hodgkin cells. (v) They show constitutive activation of numerous signaling pathways. Recent studies have begun to uncover the basis of these specific features of cHL: HRS cells actively orchestrate their complex microenvironment and attract many distinct subsets of immune cells into the affected tissues, to support their survival and proliferation, and to create an immunosuppressive environment. Reed-Sternberg cells are generated by incomplete cytokinesis and refusion of Hodgkin cells. Epstein-Barr virus (EBV) plays a major role in the rescue of crippled GC B cells from apoptosis and hence is a main player in early steps of lymphomagenesis of EBV^+^ cHL cases. The analysis of the landscape of genetic lesions in HRS cells so far did not reveal any highly recurrent HRS cell-specific lesions, but major roles of genetic lesions in members of the NF-κB and JAK/STAT pathways and of factors of immune evasion. It is perhaps the combination of the genetic lesions and the peculiar cellular origin of HRS cells that are disease defining. A combination of such genetic lesions and multiple cellular interactions with cells in the microenvironment causes the constitutive activation of many signaling pathways, often interacting in complex fashions. In nodular lymphocyte predominant Hodgkin lymphoma, the GC B cell-derived tumor cells have largely retained their typical GC B-cell expression program and follicular microenvironment. For IgD-positive cases, bacterial antigen triggering has recently been implicated in early stages of its pathogenesis.

## Introduction

Hodgkin lymphoma (HL) is one of the most frequent lymphomas in the western world, with an incidence of about 3 new cases per 100,000 individuals per year. It has a peculiar age distribution with a prevalence not only in elderly persons, but also in young adults, making it one of the most frequent cancers overall in this age group. HL is among the best treatable lymphomas, showing cure rates of about 80–90% with combined chemo-/radiotherapy [1]. New treatment approaches, in particular with immune checkpoint inhibitors and drug-conjugated anti-CD30 antibodies, show promising results in improving cure and reducing side effects and long-term toxicity associated with conventional therapy [2].

HL is divided in two main forms, classical HL (cHL) and nodular lymphocyte predominant HL (NLPHL) [3]. The tumor cells are termed Hodgkin and Reed-Sternberg (HRS) cells in cHL and lymphocyte predominant (LP) cells in NLPHL. These forms of HL differ in the morphology and immunophenotype of the lymphoma cells, the composition of the lymphoma microenvironment, and their clinical behavior [3].

Whereas treatment of HL is a major success story, and although HL is the first lymphoid malignancy that was recognized, with a first description more than 150 years ago [4], its biology and pathogenesis have been enigmatic for a long time. A main reason for this is the rareness of the tumor cells in the affected lymph nodes, which very much hindered their molecular analysis. However, major advances in our understanding of HL biology were made in the last few decades, which are discussed in this review.

## Cellular origin of HRS and LP Cells

**Fig. 1:**
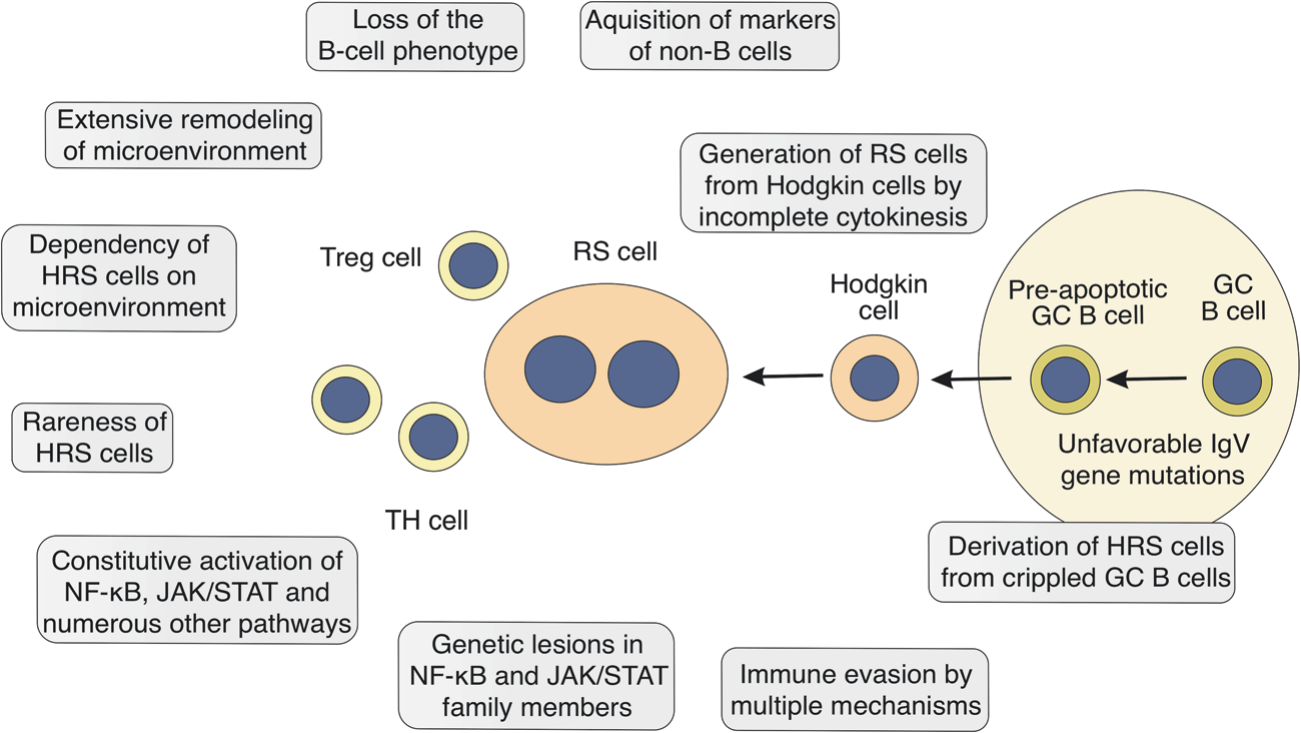
Hallmarks of HRS cells and cHL. Shown are hallmark features of HRS cells and the microenvironment in cHL.

The cellular origin of HRS cells has been unclear for a long time, because these cells show an immunophenotype that does not fit to any normal type of immune cells. HRS cells show a puzzling co-expression of markers of various cell types of the hematopoietic system. Expression of the B-cell transcription factor PAX5 pointed to a B-cell origin [5], but lack of B-cell receptor (BCR) expression and of numerous other B-cell markers argued against a B-cell identity [6, 7]. Only through genetic analysis of isolated HRS cells it was clarified that HRS cells represent transformed B cells, because these cells carry immunoglobulin (Ig) heavy and light chain V gene rearrangements, which are specific for B cells [8–11]. Moreover, the detection of somatic mutations in the IgV genes demonstrated a derivation from germinal center (GC)-experienced B cells, as the process of somatic hypermutation that generates such mutations takes place exclusively in GC B cells [8, 9, 12]. GC are the histological structures in secondary lymphoid organs in which T cell-dependent humoral immune responses take place. The detection of destructive IgV gene mutations (e.g., nonsense mutations) in a quarter of HL cases furthermore pointed to an HRS-cell derivation from pre-apoptotic GC B cells [8, 9, 12], because GC B cells acquiring such mutations normally undergo apoptosis (Fig. 1). As many disadvantageous mutations cannot be easily identified, HRS cells likely originate from pre-apoptotic GC B cells as a rule.

It should be noted that a few cases with a diagnosis of cHL are of T-cell origin [13, 14]. Whether T cell-derived HL indeed exists, or whether these are bone fide T-cell lymphomas mimicking morphologically and histologically cHL is an unresolved issue. This is also not easy to resolve, as T-cell marker expression is also seen by B cell-derived HRS cells [13, 14], so a technically demanding genetic single-cell analysis is needed to identify putative T-cell HL cases. Notably, in a global gene expression profiling study, the T cell-derived HL cell line HDLM2 clustered more closely to B cell-derived HL cell lines than to cell lines of the T cell-derived CD30^+^ anaplastic large cell lymphoma [15]. This suggests that T cells can principally acquire an HRS cell-typical gene expression program.

The prominent expression of CD30 by HRS cells [3] prompted an analysis of a potential link of HRS cells to normal CD30^+^ B cells, which are present in tonsils and lymph nodes at very low frequency inside and also outside of GCs. Most normal CD30^+^ GC B cells have mutated IgV genes, are class-switched, and express a strong MYC signature as well as MYC protein [16]. All these features are hallmarks of HRS cells, too. Moreover, in their global gene expression pattern, HRS cells are more similar to normal CD30^+^ B cells than to bulk GC B cells, memory B cells or plasma cells, pointing to a close relationship of these cells, although CD30^+^ B cells express a B-cell program that is largely lost by HRS cells [16]. However, normal CD30^+^ GC B cells presumably represent positively selected light zone GC B cells that return to the dark zone of the GC for a further round of proliferation and mutation. A possible scenario is that HRS cells develop from apoptosis-prone GC B cells that managed to escape the apoptosis program by acquiring some key features of CD30^+^ B cells, including MYC, NF-κB, and JAK/STAT pathway activities.

The sequence of events during malignant transformation of pre-apoptotic GC B cells toward HRS cells is poorly understood, but escape from programmed cell death seems to be an early and essential event. In this regard, it is an intriguing observation that all cases with crippling mutations that prevent expression of a BCR were found to be Epstein-Barr virus (EBV) positive [17]. In general, about 30% of cHL cases in the western world show a latent infection of HRS cells by EBV [18]. In EBV^+^ cases, besides a few other viral genes, two latent membrane proteins are expressed, LMP1 and LMP2a [18]. LMP1 mimics an active CD40 receptor, and LMP2a mimics BCR signaling, which are the main survival signals for GC B cells [18]. Indeed, LMP2a can rescue BCR-crippled GC B cells from apoptosis [19]. Therefore, in a fraction of cHL cases, EBV seems to play a major role in early steps of the transformation process toward HRS cells.

The mechanisms for the downregulation of B-cell program in HRS cells are not well understood, but a number of contributing factors are known. This includes downregulation of transcription factors for B-cell genes (e.g., OCT2, PU.1, BOB1), upregulation of transcription factors that suppress B-cell gene expression (e.g., ID2, ABF1, NOTCH1, STAT5), and epigenetic silencing of B-cell genes (reviewed in [12]).

In NLPHL, the phenotype of the LP cells indicated their GC B-cell origin, as they express general B-cell markers (e.g., CD19, CD20, CD79) and GC B cell-associated molecules (e.g., BCL6, HGAL, and activation-induced cytidine deaminase) [20, 21]. LP cells carry somatically mutated IgV genes, also genetically demonstrating their GC experience [22, 23]. Partly ongoing somatic hypermutation as well as their global gene expression profile supports the GC B-cell origin of LP cells [22–24]. LP cells express their BCR, and the mutation pattern indicates selection for expression of a functional BCR [22]. A direct role of antigen triggering in the pathogenesis of NLPHL is supported by the finding that in IgD-expressing LP cells, the BCR frequently binds to a *Moraxella catarrhalis*-derived antigen and the M. catarrhalis encoded IgD-binding superantigen MID/hag, causing an unusual combined antigenic and superantigenic triggering of the BCR of LP cells [25].

## Landscape of genetic lesions in HRS and LP cells

CHL is one of the lymphoid malignancies with the largest extent of chromosomal abnormalities. Aneuploidy is seen in nearly all cases, and structural aberrations and gains and losses are also common [26]. These events can also be subclonal, indicating that HRS cells have a high genomic instability. Translocations affecting the Ig loci are seen in about 20% of cases [27], but the translocation partners are diverse, and as the Ig loci are silenced in HRS cells, the role of these translocations in the established lymphoma clone remains largely unclear. The reasons for the frequent numerical and structural chromosomal aberrations are still mostly unknown. Telomere dysfunction is likely involved in these processes [28].

A particular genetic feature of cHL is that a fraction of the lymphoma clone (the Reed-Sternberg cells) is bi- or multinucleated. The generation of these cells from the mononuclear Hodgkin cells happens as a consequence of incomplete cytokinesis and refusion of daughter cells (Fig. 1) [29]. The causes for the consistent appearance of bi-/multinucleated cells in HL are not understood, but a downregulation of key factors of the cytokinesis process may contribute to this [16]. Notably, studies with HL cell lines indicate that the mononuclear Hodgkin cells are the proliferative compartment of the lymphoma clone, whereas Reed-Sternberg cells have little further proliferative potential [29, 30]. Reed-Sternberg cells are nevertheless likely important for the pathophysiology of the tumor, as they contribute to shaping the microenvironment in a lymphoma-supporting manner.

The search for somatic mutations in specific genes in HRS cells was hampered by the need to isolate the rare HRS cells for molecular analysis by microdissection from tissue sections or flow cytometric cell sorting from suspensions of viable cells. Therefore, relatively few genes were studied for mutations, and only recently the first whole exome sequencing studies began to provide a more in-depth picture of the landscape of genetic lesions in HRS cells [31–33]. A main finding from numerous studies is that mutations in members of the NF-κB pathway are a main feature of HRS cells (Table 1). This includes gains and amplifications of the genes encoding the NF-κB factor REL, the kinase MAP3K14 (also known as NIK), and BCL3 [34–38]. Inactivating mutations in the genes *TNFAIP3*, *NFKBIA*, and *NFKBIE*, encoding negative NF-κB regulators, are also frequent [39–42]. Less frequent are genetic lesions in other negative regulators of NF-κB, namely inactivating mutations or deletions of *CYLD* and *TRAF3* [43, 44]. Notably, *TNFAIP3* and *NFKBIA* mutations are more frequent in EBV-uninfected cHL, indicating that in EBV-positive cases the viral LMP1, a strong NF-κB activator, can replace the need to inactivate *TNFAIP3* or *NFKBIA* [40, 45]. As is obvious from the list of mutated NF-κB pathway factors, both the canonical and the non-canonical NF-κB pathways are affected by mutations in HRS cells. Moreover, mainly based from studies of HL cell lines, it seems that often multiple of such factors show mutations in the same HRS-cell clones [34]. This suggests that more than one lesion is often needed to dysregulate the NF-κB pathway sufficiently.

**Table 1:** Genetic lesions in HRS and LP cells.

Gene	Pathway or main function	Type of genetic alteration	Approximate frequency (%)	References
HRS cells				
* NFKBIA*	NF-κB pathway	SNVs, indels	10–20	[33, 39, 45]
* NFKBIE*	NF-κB pathway	SNVs, indels	10	[32, 33, 41, 51]
* TNFAIP3*	NF-κB pathway	SNVs, indels	40	[40, 42]
* REL*	NF-κB pathway	Gains/amplifications	50	[35, 36, 38]
* MAP3K14*	NF-κB pathway	Gains/amplifications	25	[38, 43]
* BCL3*	NF-κB pathway	Gains, translocations	20	[37]
* JAK2*^a^	JAK/STAT pathway	Gains/amplification	30	[48, 52]
* SOCS1*	JAK/STAT pathway	SNVs, indels	40	[31–33, 47]
* STAT6*	JAK/STAT pathway	SNVs, gains	30	[32, 50, 51]
* PTPN1*	JAK/STAT pathway	SNVs, indels	20	[32, 33, 46]
* CSF2RB*	JAK/STAT pathway	SNVs	20	[31, 33]
* ITPKB*	PI3K/AKT pathway	SNVs	15	[31, 32, 51]
* GNA13*	PI3K/AKT pathway	SNVs	20	[31–33, 51]
* B2M*	Immune evasion	SNVs, indels	30	[31–33, 51]
* MHC2TA*	Immune evasion	Translocations, SNVs	15	[51, 54]
* PD-L1, PD-L2*^a^	Immune evasion	Gains/amplifications	30	[49, 52, 53]
* XPO1*	Nuclear RNA and protein export	SNVs (codon 571), gains	20	[32, 33, 56]
* ARID1A*	Chromatin remodeling	SNVs, indels	25	[33]
* JMJD2C*^a^	Epigenetic regulator	Gains/amplifications	30	[49]
LP cells				
* BCL6*	Transcription factor	Translocations	35	[58]
* SOCS1*	JAK/STAT pathway	SNVs, indels	40	[61]
* SGK1*		SNVs	50	[62]
* JUNB*	Transcription factor	SNVs	50	[62]
* DUSP2*		SNVs	50	[62]
* REL*	NF-κB pathway	Gains	40	[60]

*SNV* single nucleotide variants, i.e., somatic point mutations.

^a^*PD-L1*, *PD-L2*, *JAK2*, and *JMJD2C* are located close to each other and hence mostly co-gained or co-amplified.

The JAK/STAT signaling pathway, which is constitutively active in HRS cells and represents the main mediator of cytokine signaling, is a second pathway that shows recurrently mutated genes in various of its members in HRS cells (Table 1). Besides frequent gains of the *JAK2* gene, inactivating mutations in the two main negative JAK/STAT regulators SOCS1 and PTPN1 are frequent in HRS cells [46–49]. Gains of the *STAT6* gene or activating mutations that cluster in its DNA-binding domain were detected in about one-third of cHL cases, whereas *STAT5* and *STAT3* are mutated at lower frequency [32, 33, 50]. Mutations in the *CSF2RB* gene, which codes for the common chain of CSF, IL3, and IL5 receptors, likely support constitutive JAK/STAT signaling [31, 33].

Mutations in *ITPKB*, encoding inositol-trisphosphate 3-kinase B, are detected in 13–40% of cases [31, 32, 51]. This kinase likely promotes constitutive AKT signaling. Disruptive mutations in the *GNA13* gene encoding G protein subunit alpha-13 may further contribute to constitutive AKT activity in HRS cells by disrupting receptor signaling, e.g., through S1PR2 and P2RY8 [31–33]. *GNA13* mutations were mainly identified in EBV-negative cHL [33]. In EBV-infected HL, LMP2a is expressed and may mimic BCR signaling, thereby promoting PI3K/AKT activity in these cases.

A further group of recurrent genetic lesions mainly affects immune evasion of HRS cells (Table 1). Copy number gains or amplifications including the genes *PD-L1* and *PD-L2* at 9p24.1 are among the most frequent genetic lesions in HRS cells, seen in about 75% of cases [52, 53]. Their overexpression by HRS cells provides a major immunosuppressive mechanism since by binding to PD1, they inhibit the activity of cytotoxic T cells and other PD1-expressing immune cells. Further genetic immune evasion strategies of HRS cells include frequent inactivating mutations in *B2M* that impair MHC class I expression and thereby recognition of HRS cells by CD8^+^ T cells [31] and aberrations of the *CIITA* gene that impair MHC class II expression in some cases [54]. Deletions or inactivating mutations have also been found recurrently for the *CD58* gene [55]. Whereas CD58 may on the one hand promote an HRS cell-supporting interaction with surrounding CD4^+^ T cells, its loss on the other hand is advantageous for HRS cells to escape from an attack by natural killer (NK) cells.

Additional genes that are frequently affected by genetic lesions in HRS cells are *ARID1A*, *TNFRS14*, and *XPO1* (Table 1) [33, 56, 57]. ARID1A is part of a chromatin remodeling complex and a tumor suppressor in various types of solid and hematological malignancies. *TNFRSF14* is affected by focal deletions in about 20% of cHL [33, 57]. This member of the tumor necrosis factor receptor (TNFR) superfamily is frequently mutated in various forms of B-cell lymphomas, and the inactivation of this inhibitory receptor promotes B-cell proliferation and GC B-cell survival. Exportin 1 (XPO1) is a nuclear export receptor that mediates translocation of various RNAs and proteins from the nucleus into the cytoplasm. Most mutations in *XPO1* affect codon 571 (E571K), pointing to a gain of function effect of the mutation [56]. In addition, focal gains of the *XPO1* gene have been identified [33].

Although it is likely that additional recurrent genetic lesions will be identified in further studies, a number of conclusions can already be drawn. First, no genetic lesion is found in practically all of the cases, so a disease-defining lesion (such as *MYC* translocations in Burkitt lymphoma or *BRAF* mutations in hairy cell leukemia) is missing. Second, it indeed appears that it is not specific genes that are essential for HL pathogenesis, but the dysregulation of particular pathways, which can occur through mutations affecting various pathway members. The genetic lesions in members of the NF-κB and JAK/STAT pathway are the most impressive examples in this regard, likely affecting nearly all cases. Third, extending the prior observation of *TNFAIP3* and *NFKBIA* mutations mostly in EBV-negative cases of HL (see above), two exome sequencing studies of HRS cells indicate that EBV-infected HRS-cell clones have a substantially lower mutation load than EBV-negative cases [32, 33]. This indicates that the expression of viral genes substitutes for the need of many oncogene and tumor suppressor gene mutations and thereby further supports a pathogenetic role of EBV in EBV^+^ HL. Fourth, so far, no frequent genetic lesion has been identified that could explain the loss of the B-cell gene expression program of HRS cells. Fifth, no genetic lesions have been identified that are specific for HRS cells. Nearly all genes frequently mutated in HRS cells have also been found mutated in other B-cell malignancies. It is perhaps the combination of genetic alterations that is specific for cHL. Moreover, one may speculate that the identity of HRS cells and the uniqueness of cHL is not solely defined by the landscape of its genetic lesions, but by the fact that the transforming events (partly) occurred in a very peculiar cell, namely a pre-apoptotic GC B cell.

In NLPHL, only a few recurrent genetic lesions are known (Table 1). LP cells frequently carry *BCL6* translocations that often involve Ig loci as translocation partner [58]. Similar to HRS cells, LP cells frequently show gains of *REL* and constitutive NF-κB pathway activity, but genetic lesions involving *TNFAIP3* or *NFKBIA* are absent or at least rare in LP cells [24, 59, 60]. As LP cells also lack EBV infection, the mechanisms for constitutive NF-κB activity seem to be largely distinct in the two forms of HL. Mutations in *SOCS1* were found in about 50% of NLPHL cases, implying an important role of constitutive JAK/STAT activity also in this form of HL [61]. A targeted sequencing analysis of LP cells identified the *SGK1*, *DUSP2*, and *JUNB* genes to be each recurrently mutated in approximately half of the cases analyzed [62]. Variants in the *SGK1* gene have also been found in other GC B cell-derived lymphomas [63]. This kinase plays an important role in cellular stress responses, and it has been associated with various functions, e.g., survival and proliferation, and seems to trigger the NF-κB and MAPK pathways. However, the role of SGK1 in the pathobiology of NLPHL remains unclear. Likewise, dual specificity phosphatase 2 (DUSP2) negatively regulates different pathways including the activities of MAPK, ERK, and JNK. Disrupting *DUSP2* mutations are therefore likely to contribute to the constitutive activity of these pathways in LP cells. High constitutive activity of AP-1 family members including JUNB is a hallmark of HRS cells [64, 65], but in LP cells, *JUNB* mutations seemingly support a tumor suppressor role [62]. Overall, the landscape of genetic lesion of LP cells remains to be fully explored, but seems to be remarkably distinct from the pattern of mutated genes in cHL.

## Complex pattern of deregulated signaling pathways and transcription factors in HRS cells

Tumor cells frequently hijack signaling pathways and transcription factor networks that are typically used in their normal cellular counterparts. HRS cells engage multiple signaling pathways and downstream associated transcription factors that play central roles in B-cell activation, but in contrast to numerous non-HLs, BCR signaling is not involved in cHL pathophysiology, as HRS cells lack BCR expression. Various genetic lesions profoundly contribute to the deregulation of signaling pathways and transcription factor networks, as already discussed above. Moreover, many pathways are also engaged in an autocrine and/or paracrine fashion to support HRS-cell pathobiology. HRS cells depend on the aberrant, constitutive activity of several prominent signaling pathways, such as NF-κB, JAK/STAT, and PI3K/AKT pathways (Fig. 1).

High constitutive activity of the NF-κB pathway is a hallmark of HRS cells and studies with HL cell lines revealed that this activity is essential for HRS-cell survival [34, 66]. The constitutive NF-κB activity is achieved not only by various genetic lesions, as already outlined (Table 1), but also by signaling through numerous receptors. Several TNFR family members transmit pro-survival, anti-apoptotic, and pro-proliferative signals via NF-κB, such as CD30 and CD40 [34]. Mast cells and eosinophils reportedly express CD30 ligand (CD153), and CD40 ligand is expressed by T cells that are often present in close proximity to HRS cells [67, 68]. Apart from CD40, the expression of TNFR family members BCMA, TACI, and RANK is preserved in HRS cells, which may further contribute to the high activity of NF-κB in these cells [34]. The distinct functional roles of these molecules, and their individual and/or complementary contribution are incompletely defined. Although CD30 is consistently and strongly expressed by HRS cells, there is a controversial discussion about the role of CD30 in NF-κB activation in HRS cells [69, 70]. Following ligand-induced oligomerization of the corresponding TNFR family members, TNF receptor activated factors (TRAFs) act as proximal intracellular mediators and activate IKK complexes [34]. HRS cells express TRAF1, TRAF2, and TRAF5. In EBV-positive HRS cells, the LMP1 protein mimics CD40 signal transduction and can autonomously contribute to NF-κB activation [71]. Whereas normal B cells (including GC B cells) as well as several types of B-cell non-HLs typically show predominant activation of either the canonical or the non-canonical NF-κB pathway (which is transient in the case of normal B cells), HRS cells show strong concomitant activity of both pathways, further supporting a major role of this pathway in HL pathogenesis [71, 72].

Cytokine signaling through the JAK/STAT pathway plays a key role in regulating B-cell activation and differentiation in immune responses. Constitutive cytokine expression and JAK/STAT activity have long been recognized and represent a well-known hallmark of HRS cells. The secretion of numerous interleukins (such as IL5, IL6, IL7, IL13, IL15, and IL21) as well as expression of their receptors by HRS cells supports the idea that they can function in an autocrine manner [73–76]. However, also infiltrating cells in the HL microenvironment contribute to JAK/STAT signaling in HRS cells in a paracrine fashion. At the same time, HRS cells frequently carry genetic lesions in components of the JAK/STAT pathway, as discussed above (Table 1). Several STATs (STAT3, STAT5, and STAT6) are constitutively active in HL, and promote survival and proliferation of the tumor cells [75, 77, 78]. STAT5a also reportedly contributes to the downregulation of B-cell markers in HRS cells [75]. STAT activities can also support other potentially oncogenic factors, such as AP-1 factors including BATF3 [64]. AP-1/BATF3 enhance the expression of MYC in HRS cells [64], which complements the activity of JAK2 on histones at the *MYC* locus, keeping chromatin open and accessible.

For the survival of mature B cells, tonic BCR signaling via the PI3K/AKT kinases is essential. In normal GC B cells PI3K/AKT signals contribute to the downregulation of the dark zone B-cell program. The activity of transcription factor FOXO1, which coregulates the dark zone program, is inhibited upon phosphorylation by AKT [79, 80]. In HRS cells, phosphorylated, active AKT has been detected in the majority of cHL cases analyzed, in line with the observation that PI3K isoforms are frequently expressed by HRS cells [81]. PI3K/AKT inhibition induces cell cycle arrest and apoptosis in HL cell lines [81]. PI3K/AKT activity in HRS cells also inhibits FOXO1, which acts as tumor suppressor in cHL [82]. In the absence of a BCR, several other receptor molecules have been reported to promote constitutive PI3K/AKT signaling in HRS cells, including CD30, CD40 and RANK. As mentioned earlier, also some genetic lesions in HRS cells promote AKT activity. A further mechanism for AKT activation in HRS cells is likely mediated through G protein coupled receptors. HRS cells frequently express S1PR1, and its ligand S1P induces AKT phosphorylation in HL cell lines [83]. Moreover, the *GNA13* gene encoding the G protein G13α subunit is mutated in approximately 25% of HL cases [31–33]. Mutations in *GNA13* lead to increased phospho-AKT levels and enhance AKT signaling in B-cell lymphoma cell lines [84].

Immunohistochemical analyses revealed that several receptor tyrosine kinases (RTKs) are expressed in up to 75% of cHL cases analyzed, including PDGFRA, DDR1, DDR2, EPHB1, TRKA, RON, CSF1R, and MET [85–88]. Their expression can be considered as aberrant, because most of them are not expressed by normal GC B cells [85, 86]. As they are also not expressed by other B-cell lymphomas, their expression is largely HL-specific among B-cell malignancies. Mutations in any of these molecules have not been identified, but for some of them, their physiological ligands are expressed in the HL microenvironment or by the HRS cells themselves, implying ligand-mediated stimulation [85–87, 89]. Notably, expression of the myeloid RTK CSF1R in HRS cells is mediated by aberrant derepression of an endogenous long terminal repeat located near the gene [87], and there is indication that numerous genes in HRS cells are aberrantly expressed due to reactivation of endogenous retroviral sequences [87, 90]. The precise function and relevance of RTKs in HL pathogenesis remain largely unclear, but some (e.g., TRKA, CSF1R) may support cell proliferation [87, 91]. The aberrant activity of the RTKs may influence several key pathways in HL, as RTKs are known to signal through the JAK/STAT, PI3K/AKT, and MAPK/ERK pathways. Indeed, also the MAPK/ERK pathway is constitutively active in HRS cells, with active ERK1, ERK2, and ERK5 detectable in a large fraction of HL cases [92]. Inhibition of ERK activity in HL cell lines has anti-proliferative effects, supporting a functional role of MAPK/ERK signaling in HL pathophysiology [92].

Several signaling pathways contribute to activation of members of the AP-1 transcription factor family in HRS cells. Overexpression of JUNB is mediated by NF-κB signaling, and BATF3 expression is induced by STAT3 and STAT6 [64, 65]. Various combinations of homo- and heterodimers of AP-1 family members JUN, JUNB, ATF3, and BATF3 have been detected in HRS cells [64, 65]. Target genes of AP-1 in HRS cells include CD30 and galectin-1, which has immunosuppresive functions [93, 94]. AP-1 heterodimers of BATF3 with JUN or JUNB bind to the *MYC* gene and induce MYC expression in HRS cells [64]. An essential role of BATF3 activity for HRS cells was demonstrated by showing a toxic effect of its experimental downregulation in HL cell lines [64].

A further constitutively active signaling pathway in HRS cells is the NOTCH1 pathway [95]. NOTCH1 is highly expressed by HRS cells, and its ligand JAGGED1 is provided by cells in the microenvironment and by HRS cells themselves [95]. In HL cell lines, NOTCH1 signaling promotes HRS cell survival and proliferation [95].

Taken together, HRS cells are characterized by the deregulated and partly aberrant constitutive activation of multiple signaling pathways and transcription factors. A complex transcription factor network emerges from these signaling pathways and their interconnection. The extent to which many signaling pathways are constitutively activated seems to be quite unique for cHL among B-cell malignancies.

## Microenvironmental interactions

The microenvironment of non-tumor cells in cHL represents the main tumor mass and is another very characteristic feature of this disease (Fig. 1). HRS cells typically account for only about 1% of cells in the lymphoma tissue. CHL has the most heterogenous cellular composition among lymphomas, consisting of various innate (e.g., eosinophils, neutrophils, mast cells, macrophages, NK cells) and adaptive (B cells, plasma cells, CD4^+^ helper (Th) and regulatory (Treg) T cells, CD8^+^ T cells) immune cell types, stroma cells, and fibroblasts. Each of the four histological subtypes (nodular sclerosis, mixed cellularity, lymphocyte-rich, lymphocyte depleted) has particular patterns, such as numerous fibroblast-like cells and dominant fibrosis in nodular sclerosis HL as an example [3]. Among these dominant non-malignant cells, HRS cells are widely scattered. CD4^+^ T cells typically represent the largest population of non-malignant immune cells in the cHL microenvironment [3].

**Fig. 2:**
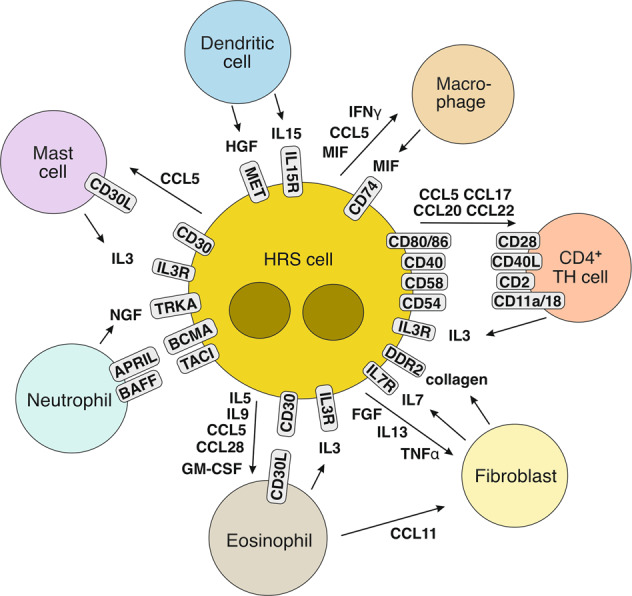
Microenvironmental interactions in cHL supporting HRS-cell survival and proliferation. Shown are main cellular interactions in the cHL microenvironment that promote the survival and/or proliferation of HRS cells. The HRS cell-supporting immune cells and fibroblasts are actively recruited into the microenvironment by a multitude of cytokines and chemokines secreted by the HRS cells. HRS cells in many cHL cases still express MHC class II, so that in addition to the interactions between HRS cells and CD4^+^ Th cells shown also a binding of MHC class II to the TCR is possible (not depicted).

The signaling pathways and transcription factors that have gained constitutive activity in HRS cells contribute a specific secretory profile of various growth factors, chemokines, and cytokines that together foster the formation of the microenvironment in HL, including remodeling of stroma and matrix cells, and migration, activation, and functions of leukocytes. Thus, HRS cells systematically shape their microenvironment, adjusting surrounding cells and recruiting various immune cell types (Fig. 2). HRS cells produce a plethora of cytokines, chemokines, and interleukins apart from several growth and stimulating factors, so their secretion supports the recruitment of different subtypes of immune cells into the tissue. CCL5 (RANTES), CCL17 (TARC), CCL20, and CCL22 produced by HRS cells are the main factors that attract CCR3-, CCR4-, and/or CCR6-expressing immune cells, including Th2 and Treg cell subsets [96–98]. These chemokines in combination with several interleukins, such as IL5, IL8, and/or IL9, also promote the recruitment of macrophages, basophils, eosinophils, and neutrophils. The production of CXC chemokine ligands, such as CXCL9 and CXCL10, by HRS cells supports the attraction of Th1 cells and NK cells. Besides secretion of soluble factors, also the production of extracellular vesicles by HRS cells likely contributes to a cross-talk with distant cells in the microenvironment, including stimulation of CD30L-expressing mast cells and eosinophils by CD30^+^ extracellular vesicles [99]. Overall, cHL seems unique among lymphoid malignancies in the extent and diversity of chemokine and cytokine production of the lymphoma cells, and the complexity of the microenvironmental reshaping (Fig. 1).

HRS cells do not only shape their microenvironment by attracting immune cells into their microenvironment, but they also modulate the function and differentiation of cells through secretion of critical factors. For example, HRS cells can induce a Treg phenotype on co-cultured T cells in vitro [100, 101]. This is likely mediated by factors secreted by HRS cells and known to induce functional reprogramming of tumor-infiltrating T cells into Th2 cells and Tregs, such as galectin-1 and IL7 [76, 93].

The extensive remodeling of the lymphoma microenvironment in cHL has most likely two main pathobiological functions, namely first to attract immune cells that support the survival and proliferation of HRS cells, and second to generate a microenvironment in which HRS cells can escape from anti-tumor immune control.

Numerous findings support the idea that HRS cells are highly dependent on their microenvironment, for example, the difficulties to grow primary HRS cells in culture or in immunodeficient mice and establish cell lines from these cells, the virtual absence of HRS cells in the peripheral blood, and the consistent presence of accompanying immune cells in HL metastases outside of lymph nodes. The low incidence of cHL in AIDS patients with low CD4^+^ T-cell counts and the increased frequency of this lymphoma in HIV^+^ patients under anti-retroviral therapy with normalized CD4^+^ T-cell counts also argue for an important role of CD4^+^ T cells in HL pathogenesis [102]. HRS cells are directly surrounded by CD4^+^ T cells, a phenomenon called rosetting [103]. T cells in close proximity to HRS cells reportedly lack CD26 expression, which has proteolytic activity and can inactivate various chemokines, including CCL5 and CCL22, and thereby probably modulate immune cell attraction [104]. CD26 downregulation is untypical on activated T cells and may be the consequence of the cellular and cytokine microenvironment in cHL, where it is likely to sustain chemokine gradients and immune cell recruitment into the tumor tissue. CD4^+^CD26^–^ T cells may derive from Treg cells and seem functionally unresponsive [104]. The tight binding and interaction of HRS cells with the surrounding T cells involves several adhesion molecules (e.g., CD54 on HRS cells and CD11a/CD18 on T cells), and also costimulatory molecules, in particular CD40, CD80, and CD86 on HRS cells and their ligands CD40L and CD28 on the rosetting T cells (Fig. 2) [103]. HRS cells in most cases of cHL still express MHC class II, so that also its interaction with the T-cell receptor can be envisioned [105], although this receptor seems to be often occupied by MHC class II-associated invariant chain peptides, indicating deficient loading of MHC class II with antigenic peptides [106]. It is remarkable that HRS cells, which have downregulated most of their B-cell gene expression program [6], still retain expression of key factors for an interaction with T helper cells. This supports the idea that signaling through costimulatory factors such as CD40 and CD80 or receptors for soluble factors secreted by the rosetting T cells (e.g., IL3) plays an important role in promoting the survival and/or proliferation of HRS cells. Discrepant findings on the predominant subtype of the CD4^+^ T helper cells in cHL—a Th2 versus Th1 subset predominance—have been reported, requiring further investigation [107]. Earlier, largely immunohistochemistry-based analyses of multiple T-cell differentiation markers pointed to a Th2 predominance in cHL [108]. A recent single-cell RNA-seq of cHL tissues also detected a major Th2 subset among Th cells, yet Th1 cells were also enriched in the cHL samples in comparison to reactive lymph nodes [100]. Other studies, mainly based on flow cytometry and CyTOF analysis, however, argued for a Th1 cell predominance in cHL [107, 109]. Besides CD40, further TNFR superfamily members may also contribute to stimulate HRS cells and promote their survival, including CD30L expressed by mast cells and eosinophils, binding to CD30 expressed by HRS cells, and APRIL on neutrophils binding to BCMA on HRS cells (Fig. 2) [103]. Furthermore, several RTKs mostly aberrantly expressed by HRS cells can be activated by their ligands which are secreted by other cells in the HL microenvironment, including HGF secreted by dendritic cells (binding to MET), NGF secreted by neutrophils (binding to TRKA), and collagen produced by fibroblasts (binding to DDR1 and DDR2) [85, 86, 91].

**Fig. 3:**
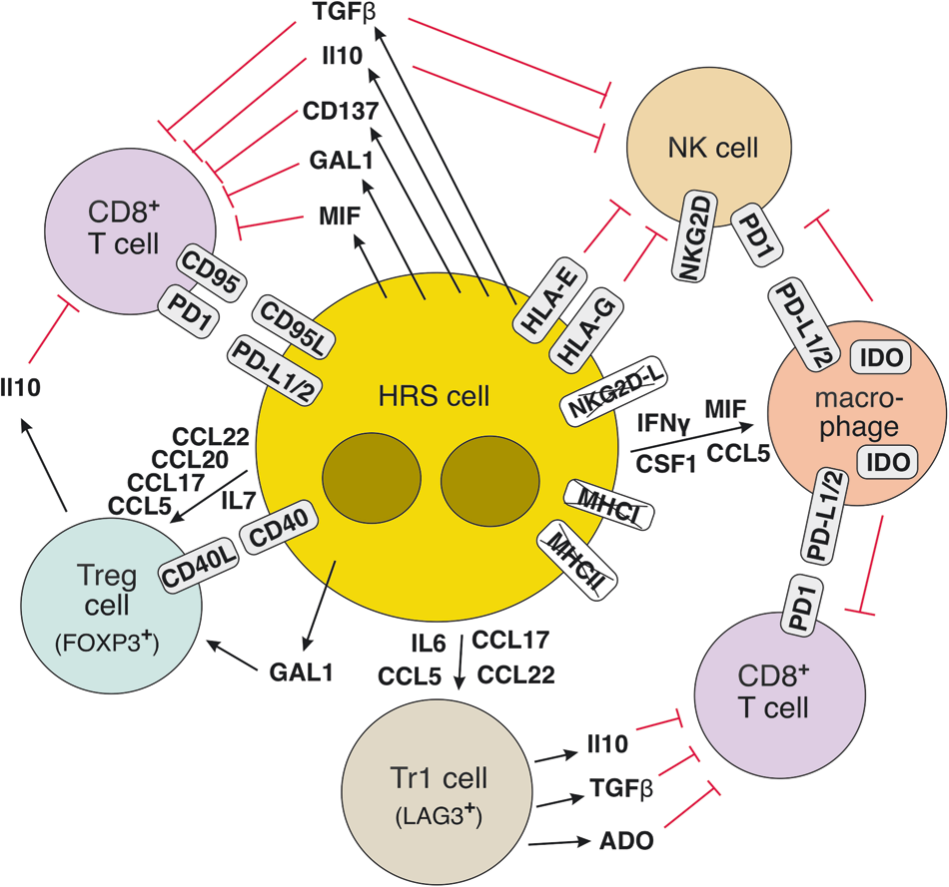
Immune evasion mechanisms in cHL. Shown are main mechanisms how HRS cells escape from an attack by cytotoxic T cells and NK cells. Several factors promote the production and reduce the degradation of extracellular adenosin (ADO), which inhibits the function of CD8^+^ T cells (see main text). Indoleamine 2,3-dioxygenase (IDO) is intracellularly expressed by macrophages and dendritic cells (not shown) in the HL microenvironment and reduces the extracellular levels of tryptophan, which impairs the function of cytotoxic T cells and NK cells. The immunosuppressive function of CD137 is thought to be mediated in an indirect fashion: CD137 on HRS cells and CD137 transmitted to other immune cells through trogocytosis promote the internalization of CD137L, thereby reducing the level of this costimulatory factor for T cell activity. The downregulation of NKG2D-L on HRS cells is mediated by exoenzymes that cleave it from the surface of HRS cells. Whereas MHC class I is downregulated by most cases of cHL, loss of MHC class II expression is seen only in a subset of cases.

As HRS cells carry a high load of somatic mutations and hence potential neoantigens, and as in EBV-positive cases viral proteins are produced by HRS cells, these lymphoma cells should principally be a target for immune control by cytotoxic cells. The cHL microenvironment indeed contains potentially cytotoxic cells, in particular CD8^+^ T cells, NK cells, and perhaps also cytotoxic CD4^+^ T cells, even though these cells represent only a minority of the cellular infiltrate. HRS cells utilize a multitude of factors that contribute to immune evasion (Fig. 3). First, HRS cells frequently lack expression of MHC class I, which is essential for a recognition by cytotoxic CD8^+^ T cells. The lack of MHC class I expression is often due to inactivating mutations in the *B2M* gene, as discussed above [31]. Lack of MHC class I expression is more frequently seen in EBV-negative than EBV-positive cases of HL [110, 111], partly due to the preferential occurrence of *B2M* mutations in EBV-negative cases [33]. Second, HRS cells secrete a number of molecules with immunosuppressive effects, in particular TGFβ, IL10, MIF (macrophage migration inhibitory factor), and galectin-1 [103, 112]. Third, HRS cells express surface molecules that inhibit cytotoxic T cells and/or NK cells, including CD95 ligand (CD95L), PD-L1, PD-L2, CD200, HLA-G, and HLA-E [52, 103, 113]. The latter two factors inhibit NK cells, whereas CD95L can induce apoptosis of CD95^+^ cytotoxic T cells. Notably, the immunosuppressive PD-L1 is not only expressed by HRS cells, but also by many macrophages in the tumor microenvironment [114], which likely contributes to the suppression of PD1^+^ immune cells. A fraction of PD-L1 detected on the surface of macrophages may derive from HRS cells themselves, transferred to macrophages though trogocytosis [115]. A further T-cell immunosuppressive factor expressed by HRS cells and potentially also transferred to other cells in the microenvironment is CD137 [116]. CD137 mediates its effect by causing internalization of CD137L, and thus withdrawal of this costimulatory signal for T-cell activation. Fourth, there is indication for immunosuppressive purinergic signaling in HL, with high microenvironmental expression of CD38, CD39, and CD73, and downregulation of CD26 on T cells, together promoting production and impairing degradation of extracellular adenosin [101]. Fifth, HRS cells attract regulatory T cells into the microenvironment [117, 118], as already mentioned above. These Treg cells can inhibit cytotoxic cells by secretion of IL10 and TGFβ, and by surface expression of CTLA-4 and LAG3 [100, 118–120]. The population of Treg cells is composed of natural FOXP3^+^ as well as LAG3^+^ Tr1 cells [100, 118–120]. Sixth, macrophages and dendritic cells in the HL microenvironment express indoleamine 2,3-dioxygenase, which reduces extracellular tryptophan levels, which is inhibitory for the function of effector T cells and NK cells [121].

Interestingly, treatment of HL patients with antibodies against PD1 or PD-L1 often results in remission [2], but the efficiency of this treatment surprisingly turned out to be correlated with MHC class II but not class I expression [105]. This indicates that the elimination of HRS cells during this immune checkpoint blockade therapy is not (mainly) mediated by re-activated cytotoxic CD8^+^ T cells, because these cells need MHC class I expression on their target cells. In support of this idea, the frequency of PD1^+^ CD8^+^ T cells is very low in HL lymph nodes, and no increase in intratumoral cytotoxic CD8^+^ T cells was observed a few days after initiation of anti-PD1 therapy, when HRS cells were already mostly eliminated [122]. Because of the link to MHC class II expression, a potential role of (cytotoxic?) CD4^+^ T cells is being discussed [105, 123]. Moreover, NK cells in HL express elevated levels of PD1, so that a release of inhibition of NK cells though PD1 ligands by the treatment can be envisioned, resulting in an attack of the MHC class I-negative HRS cells [114]. The involvement of CD4^+^ T cells and NK cells in the therapeutic anti-PD1 effect is supported by the observation that therapy success correlated with CD4^+^ T cell clonal expansions and increased numbers of activated NK cells, with the caveat that peripheral blood was studied, but not HL lymph node infiltrating immune cells [124]. A further effect of treatment of PD1-blocking antibodies could be unrelated to immune evasion, as there is indication for reverse signaling through PD-L1 in HRS cells that supports the survival and proliferation of HRS cells, and that is hence blocked by the antibody treatment [125].

Taken together, cHL shows a unique remodeling of the lymph node microenvironment, which is orchestrated by the HRS cells through secretion of numerous cytokines and chemokines. The complex microenvironment serves to support survival and proliferation of the tumor cells and to enable immune evasion of the HRS cells.

## Conclusions and perspective

In light of the various unique and peculiar features of the biology of cHL, the question arises whether there is a link between these characteristics of the disease. Considering the origin of HRS cells from pre-apoptotic GC B cells, and that GC B cells are stringently selected for expression of a high affinity BCR and cognate interaction with Th cells, one can imagine a scenario in which crippled GC B cells are under strong selective pressure to escape the B-cell gene expression program and hence the pressure to execute apoptosis. Such cells could be selected to downregulate the B-cell program and thereby the selection pressure to express a (high affinity) BCR. This idea is supported by the finding that enforced reexpression of B-cell transcription factors or a pharmacological restoration of the B-cell program induces apoptosis in HL cell lines [126–128]. For an aberrantly surviving crippled GC B cell, there is no defined gene expression program in the immune system. As all immune cells need survival signals, the putative HRS precursor cell may use the known plasticity of immune cells to upregulate a variety of other genes and signaling pathways that provide survival and proliferation signals. This is apparently achieved by a combination of genetic lesions and microenvironmental interactions. In this scenario, the lost B-cell phenotype, the upregulation of markers of other immune cells, the extensive remodeling of the microenvironment, and the activation of a uniquely complex set of signaling pathways are all linked to the derivation of HRS cells from crippled GC B cells that mange to escape the physiological apoptosis program. This is very different from other B-cell lymphomas, where there is a general scenario that lymphoma cells are frozen at a particular differentiation stage, such as follicular lymphoma and many diffuse large B-cell lymphomas at a proliferative GC B-cell stage [129]. Perhaps, the unphysiological (and partly unstable) gene expression program of HRS cells is also a reason why these fragile tumor cells are so vulnerable to chemo- and radiation therapy, explaining why cHL has such a good response to therapy.

Regarding the increasing knowledge about the genetic lesions and microenvironmental interactions and dependencies in HL, and the amazing efficiency of antibodies interfering with the PD1-PD-L1 interaction, it is to be hoped that further vulnerabilities of HRS cells can be translated into targeted treatment options. Clearly, further work is needed to fully understand the landscape of genetic lesions in HRS and LP cells, to also understand the contribution of epigenetic alterations in disease pathobiology, to clarify the mechanisms for the dramatic reprogramming of the gene program of HRS cells, and to understand why multinucleated Reed-Sternberg cells inevitably develop in cHL. The recent observation that circulating tumor DNA of HRS cells can be regularly identified in plasma at diagnosis is not only helpful to further define genetic lesions in HRS cells, but may become a very valuable tool to monitor the course of the disease under therapy [51, 130].

## References

[1] Borchmann P, Eichenauer DA, Engert A (2012). State of the art in the treatment of Hodgkin lymphoma. Nat Rev Clin Oncol.

[2] Younes A, Ansell SM (2016). Novel agents in the treatment of Hodgkin lymphoma: biological basis and clinical results. Semin Hematol.

[3] Swerdlow SH, Campo E, Pileri SA, Harris NL, Stein H, Siebert R (2016). The 2016 revision of the World Health Organization classification of lymphoid neoplasms. Blood..

[4] Hodgkin T (1832). On some morbid appearances of the absorbent glands and spleen. Med Chir Trans.

[5] Foss HD, Reusch R, Demel G, Lenz G, Anagnostopoulos I, Hummel M (1999). Frequent expression of the B-cell-specific activator protein in Reed-Sternberg cells of classical Hodgkin’s disease provides further evidence for its B-cell origin. Blood..

[6] Schwering I, Bräuninger A, Klein U, Jungnickel B, Tinguely M, Diehl V (2003). Loss of the B-lineage-specific gene expression program in Hodgkin and Reed-Sternberg cells of Hodgkin lymphoma. Blood..

[7] Tiacci E, Döring C, Brune V, van Noesel CJ, Klapper W, Mechtersheimer G (2012). Analyzing primary Hodgkin and Reed-Sternberg cells to capture the molecular and cellular pathogenesis of classical Hodgkin lymphoma. Blood..

[8] Bräuninger A, Wacker HH, Rajewsky K, Küppers R, Hansmann ML (2003). Typing the histogenetic origin of the tumor cells of lymphocyte-rich classical Hodgkin’s lymphoma in relation to tumor cells of classical and lymphocyte-predominance Hodgkin’s lymphoma. Cancer Res.

[9] Kanzler H, Küppers R, Hansmann ML (1996). Rajewsky K. Hodgkin and Reed-Sternberg cells in Hodgkin’s disease represent the outgrowth of a dominant tumor clone derived from (crippled) germinal center B cells. J Exp Med.

[10] Küppers R, Rajewsky K, Zhao M, Simons G, Laumann R, Fischer R (1994). Hodgkin disease: Hodgkin and Reed-Sternberg cells picked from histological sections show clonal immunoglobulin gene rearrangements and appear to be derived from B cells at various stages of development. Proc Natl Acad Sci USA.

[11] Marafioti T, Hummel M, Foss H-D, Laumen H, Korbjuhn P, Anagnostopoulos I (2000). Hodgkin and Reed-Sternberg cells represent an expansion of a single clone originating from a germinal center B-cell with functional immunoglobulin gene rearrangements but defective immunoglobulin transcription. Blood..

[12] Küppers R, Engert A, Hansmann M-L (2012). Hodgkin lymphoma. J Clin Invest.

[13] Müschen M, Rajewsky K, Bräuninger A, Baur AS, Oudejans JJ, Roers A (2000). Rare occurrence of classical Hodgkin’s disease as a T cell lymphoma. J Exp Med.

[14] Seitz V, Hummel M, Marafioti T, Anagnostopoulos I, Assaf C, Stein H (2000). Detection of clonal T-cell receptor gamma-chain gene rearrangements in Reed-Sternberg cells of classic Hodgkin disease. Blood..

[15] Willenbrock K, Küppers R, Renne C, Brune V, Eckerle S, Weidmann E (2006). Common features and differences in the transcriptome of large cell anaplastic lymphoma and classical Hodgkin’s lymphoma. Haematologica..

[16] Weniger MA, Tiacci E, Schneider S, Arnolds J, Rüschenbaum S, Duppach J (2018). Human CD30+ B cells represent a unique subset related to Hodgkin lymphoma cells. J Clin Invest.

[17] Bräuninger A, Schmitz R, Bechtel D, Renne C, Hansmann ML, Küppers R (2006). Molecular biology of Hodgkin’s and Reed/Sternberg cells in Hodgkin’s lymphoma. Int J Cancer.

[18] Kapatai G, Murray P (2007). Contribution of the Epstein Barr virus to the molecular pathogenesis of Hodgkin lymphoma. J Clin Pathol.

[19] Mancao C, Hammerschmidt W (2007). Epstein-Barr virus latent membrane protein 2A is a B-cell receptor mimic and essential for B-cell survival. Blood..

[20] Greiner A, Tobollik S, Buettner M, Jungnickel B, Herrmann K, Kremmer E (2005). Differential expression of activation-induced cytidine deaminase (AID) in nodular lymphocyte-predominant and classical Hodgkin lymphoma. J Pathol.

[21] Küppers R (2009). The biology of Hodgkin’s lymphoma. Nat Rev Cancer.

[22] Braeuninger A, Küppers R, Strickler JG, Wacker HH, Rajewsky K, Hansmann ML (1997). Hodgkin and Reed-Sternberg cells in lymphocyte predominant Hodgkin disease represent clonal populations of germinal center-derived tumor B cells. Proc Natl Acad Sci USA.

[23] Marafioti T, Hummel M, Anagnostopoulos I, Foss HD, Falini B, Delsol G (1997). Origin of nodular lymphocyte-predominant Hodgkin’s disease from a clonal expansion of highly mutated germinal-center B cells. N Engl J Med.

[24] Brune V, Tiacci E, Pfeil I, Döring C, Eckerle S, van Noesel CJM (2008). Origin and pathogenesis of nodular lymphocyte-predominant Hodgkin lymphoma as revealed by global gene expression analysis. J Exp Med.

[25] Thurner L, Hartmann S, Fadle N, Regitz E, Kemele M, Kim YJ (2020). Lymphocyte predominant cells detect Moraxella catarrhalis-derived antigens in nodular lymphocyte-predominant Hodgkin lymphoma. Nat Commun.

[26] Weber-Matthiesen K, Deerberg J, Poetsch M, Grote W, Schlegelberger B (1995). Numerical chromosome aberrations are present within the CD30+ Hodgkin and Reed-Sternberg cells in 100% of analyzed cases of Hodgkin’s disease. Blood..

[27] Martin-Subero JI, Klapper W, Sotnikova A, Callet-Bauchu E, Harder L, Bastard C (2006). Chromosomal breakpoints affecting immunoglobulin loci are recurrent in Hodgkin and Reed-Sternberg cells of classical Hodgkin lymphoma. Cancer Res.

[28] Cuceu C, Hempel WM, Sabatier L, Bosq J, Carde P, M’Kacher R. Chromosomal instability in Hodgkin lymphoma: an in-depth review and perspectives. Cancers (Basel). 2018;10:91. 10.3390/cancers10040091 PMC5923346 29587466

[29] Rengstl B, Newrzela S, Heinrich T, Weiser C, Thalheimer FB, Schmid F (2013). Incomplete cytokinesis and re-fusion of small mononucleated Hodgkin cells lead to giant multinucleated Reed-Sternberg cells. Proc Natl Acad Sci USA.

[30] Ikeda J, Mamat S, Tian T, Wang Y, Rahadiani N, Aozasa K (2010). Tumorigenic potential of mononucleated small cells of Hodgkin lymphoma cell lines. Am J Pathol.

[31] Reichel J, Chadburn A, Rubinstein PG, Giulino-Roth L, Tam W, Liu Y (2015). Flow-sorting and exome sequencing reveals the oncogenome of primary Hodgkin and Reed-Sternberg cells. Blood..

[32] Tiacci E, Ladewig E, Schiavoni G, Penson A, Fortini E, Pettirossi V (2018). Pervasive mutations of JAK-STAT pathway genes in classical Hodgkin lymphoma. Blood..

[33] Wienand K, Chapuy B, Stewart C, Dunford AJ, Wu D, Kim J (2019). Genomic analyses of flow-sorted Hodgkin Reed-Sternberg cells reveal complementary mechanisms of immune evasion. Blood Adv.

[34] Weniger MA, Küppers R (2016). NF-kappaB deregulation in Hodgkin lymphoma. Semin Cancer Biol.

[35] Joos S, Menz CK, Wrobel G, Siebert R, Gesk S, Ohl S (2002). Classical Hodgkin lymphoma is characterized by recurrent copy number gains of the short arm of chromosome 2. Blood..

[36] Martin-Subero JI, Gesk S, Harder L, Sonoki T, Tucker PW, Schlegelberger B (2002). Recurrent involvement of the REL and BCL11A loci in classical Hodgkin lymphoma. Blood..

[37] Martin-Subero JI, Wlodarska I, Bastard C, Picquenot JM, Höppner J, Giefing M (2006). Chromosomal rearrangements involving the BCL3 locus are recurrent in classical Hodgkin and peripheral T-cell lymphoma. Blood..

[38] Steidl C, Telenius A, Shah SP, Farinha P, Barclay L, Boyle M (2010). Genome-wide copy number analysis of Hodgkin Reed-Sternberg cells identifies recurrent imbalances with correlations to treatment outcome. Blood..

[39] Jungnickel B, Staratschek-Jox A, Bräuninger A, Spieker T, Wolf J, Diehl V (2000). Clonal deleterious mutations in the IkappaBalpha gene in the malignant cells in Hodgkin’s lymphoma. J Exp Med.

[40] Schmitz R, Hansmann ML, Bohle V, Martin-Subero JI, Hartmann S, Mechtersheimer G (2009). TNFAIP3 (A20) is a tumor suppressor gene in Hodgkin lymphoma and primary mediastinal B cell lymphoma. J Exp Med.

[41] Emmerich F, Theurich S, Hummel M, Haeffker A, Vry MS, Döhner K (2003). Inactivating I kappa B epsilon mutations in Hodgkin/Reed-Sternberg cells. J Pathol.

[42] Kato M, Sanada M, Kato I, Sato Y, Takita J, Takeuchi K (2009). Frequent inactivation of A20 in B-cell lymphomas. Nature..

[43] Otto C, Giefing M, Massow A, Vater I, Gesk S, Schlesner M (2012). Genetic lesions of the TRAF3 and MAP3K14 genes in classical Hodgkin lymphoma. Br J Haematol.

[44] Schmidt A, Schmitz R, Giefing M, Martin-Subero JI, Gesk S, Vater I (2010). Rare occurrence of biallelic CYLD gene mutations in classical Hodgkin lymphoma. Genes Chromosomes Cancer.

[45] Lake A, Shield LA, Cordano P, Chui DT, Osborne J, Crae S (2009). Mutations of NFKBIA, encoding IkappaB alpha, are a recurrent finding in classical Hodgkin lymphoma but are not a unifying feature of non-EBV-associated cases. Int J Cancer.

[46] Gunawardana J, Chan FC, Telenius A, Woolcock B, Kridel R, Tan KL (2014). Recurrent somatic mutations of PTPN1 in primary mediastinal B cell lymphoma and Hodgkin lymphoma. Nat Genet.

[47] Weniger MA, Melzner I, Menz CK, Wegener S, Bucur AJ, Dorsch K (2006). Mutations of the tumor suppressor gene SOCS-1 in classical Hodgkin lymphoma are frequent and associated with nuclear phospho-STAT5 accumulation. Oncogene..

[48] Joos S, Küpper M, Ohl S, von Bonin F, Mechtersheimer G, Bentz M (2000). Genomic imbalances including amplification of the tyrosine kinase gene JAK2 in CD30+ Hodgkin cells. Cancer Res.

[49] Rui L, Emre NC, Kruhlak MJ, Chung HJ, Steidl C, Slack G (2010). Cooperative epigenetic modulation by cancer amplicon genes. Cancer Cell.

[50] Hartmann S, Martin-Subero JI, Gesk S, Husken J, Giefing M, Nagel I (2008). Detection of genomic imbalances in microdissected Hodgkin and Reed-Sternberg cells of classical Hodgkin’s lymphoma by array-based comparative genomic hybridization. Haematologica..

[51] Desch AK, Hartung K, Botzen A, Brobeil A, Rummel M, Kurch L (2020). Genotyping circulating tumor DNA of pediatric Hodgkin lymphoma. Leukemia..

[52] Green MR, Monti S, Rodig SJ, Juszczynski P, Currie T, O’Donnell E (2010). Integrative analysis reveals selective 9p24.1 amplification, increased PD-1 ligand expression, and further induction via JAK2 in nodular sclerosing Hodgkin lymphoma and primary mediastinal large B-cell lymphoma. Blood..

[53] Roemer MG, Advani RH, Ligon AH, Natkunam Y, Redd RA, Homer H (2016). PD-L1 and PD-L2 genetic alterations define classical Hodgkin lymphoma and predict outcome. J Clin Oncol.

[54] Steidl C, Shah SP, Woolcock BW, Rui L, Kawahara M, Farinha P (2011). MHC class II transactivator CIITA is a recurrent gene fusion partner in lymphoid cancers. Nature..

[55] Schneider M, Schneider S, Zühlke-Jenisch R, Klapper W, Sundström C, Hartmann S (2015). Alterations of the CD58 gene in classical Hodgkin lymphoma. Genes Chromosomes Cancer.

[56] Camus V, Stamatoullas A, Mareschal S, Viailly PJ, Sarafan-Vasseur N, Bohers E (2016). Detection and prognostic value of recurrent exportin 1 mutations in tumor and cell-free circulating DNA of patients with classical Hodgkin lymphoma. Haematologica..

[57] Salipante SJ, Adey A, Thomas A, Lee C, Liu YJ, Kumar A (2016). Recurrent somatic loss of TNFRSF14 in classical Hodgkin lymphoma. Genes Chromosomes Cancer.

[58] Wlodarska I, Nooyen P, Maes B, Martin-Subero JI, Siebert R, Pauwels P (2003). Frequent occurrence of BCL6 rearrangements in nodular lymphocyte predominance Hodgkin lymphoma but not in classical Hodgkin lymphoma. Blood..

[59] Schumacher MA, Schmitz R, Brune V, Tiacci E, Döring C, Hansmann ML (2010). Mutations in the genes coding for the NF-kappaB regulating factors IkappaBalpha and A20 are uncommon in nodular lymphocyte-predominant Hodgkin’s lymphoma. Haematologica..

[60] Hartmann S, Döring C, Vucic E, Chan FC, Ennishi D, Tousseyn T (2015). Array comparative genomic hybridization reveals similarities between nodular lymphocyte predominant Hodgkin lymphoma and T cell/histiocyte rich large B cell lymphoma. Br J Haematol.

[61] Mottok A, Renné C, Willenbrock K, Hansmann ML, Bräuninger A (2007). Somatic hypermutation of SOCS1 in lymphocyte-predominant Hodgkin lymphoma is accompanied by high JAK2 expression and activation of STAT6. Blood..

[62] Hartmann S, Schuhmacher B, Rausch T, Fuller L, Döring C, Weniger M (2016). Highly recurrent mutations of SGK1, DUSP2 and JUNB in nodular lymphocyte predominant Hodgkin lymphoma. Leukemia..

[63] Morin RD, Mendez-Lago M, Mungall AJ, Goya R, Mungall KL, Corbett RD (2011). Frequent mutation of histone-modifying genes in non-Hodgkin lymphoma. Nature..

[64] Lollies A, Hartmann S, Schneider M, Bracht T, Weiss AL, Arnolds J (2018). An oncogenic axis of STAT-mediated BATF3 upregulation causing MYC activity in classical Hodgkin lymphoma and anaplastic large cell lymphoma. Leukemia..

[65] Mathas S, Hinz M, Anagnostopoulos I, Krappmann D, Lietz A, Jundt F (2002). Aberrantly expressed c-Jun and JunB are a hallmark of Hodgkin lymphoma cells, stimulate proliferation and synergize with NF-kappa B. EMBO J.

[66] Bargou RC, Emmerich F, Krappmann D, Bommert K, Mapara MY, Arnold W (1997). Constitutive nuclear factor-kappaB-RelA activation is required for proliferation and survival of Hodgkin’s disease tumor cells. J Clin Invest.

[67] Carbone A, Gloghini A, Gruss HJ, Pinto A (1995). CD40 ligand is constitutively expressed in a subset of T cell lymphomas and on the microenvironmental reactive T cells of follicular lymphomas and Hodgkin’s disease. Am J Pathol.

[68] Molin D, Fischer M, Xiang Z, Larsson U, Harvima I, Venge P (2001). Mast cells express functional CD30 ligand and are the predominant CD30L-positive cells in Hodgkin’s disease. Br J Haematol.

[69] Hirsch B, Hummel M, Bentink S, Fouladi F, Spang R, Zollinger R (2008). CD30-induced signaling is absent in Hodgkin’s cells but present in anaplastic large cell lymphoma cells. Am J Pathol.

[70] Horie R, Watanabe T, Morishita Y, Ito K, Ishida T, Kanegae Y (2002). Ligand-independent signaling by overexpressed CD30 drives NF-kappaB activation in Hodgkin-Reed-Sternberg cells. Oncogene..

[71] Kilger E, Kieser A, Baumann M, Hammerschmidt W (1998). Epstein-Barr virus-mediated B-cell proliferation is dependent upon latent membrane protein 1, which simulates an activated CD40 receptor. EMBO J.

[72] de Oliveira KAP, Kaergel E, Heinig M, Fontaine J-F, Patone G, Muro EM (2016). A roadmap of constitutive NF-kB activity in Hodgkin lymphoma: Dominant roles of p50 and p52 revealed by genome-wide analyses. Genome Med.

[73] Kapp U, Yeh WC, Patterson B, Elia AJ, Kagi D, Ho A (1999). Interleukin 13 is secreted by and stimulates the growth of Hodgkin and Reed-Sternberg cells. J Exp Med.

[74] Lamprecht B, Kreher S, Anagnostopoulos I, Johrens K, Monteleone G, Jundt F (2008). Aberrant expression of the Th2 cytokine IL-21 in Hodgkin lymphoma cells regulates STAT3 signaling and attracts Treg cells via regulation of MIP-3{alpha}. Blood..

[75] Scheeren FA, Diehl SA, Smit LA, Beaumont T, Naspetti M, Bende RJ (2008). IL-21 is expressed in Hodgkin lymphoma and activates STAT5; evidence that activated STAT5 is required for Hodgkin lymphomagenesis. Blood..

[76] Cattaruzza L, Gloghini A, Olivo K, Di Francia R, Lorenzon D, De Filippi R (2009). Functional coexpression of Interleukin (IL)-7 and its receptor (IL-7R) on Hodgkin and Reed-Sternberg cells: Involvement of IL-7 in tumor cell growth and microenvironmental interactions of Hodgkin’s lymphoma. Int J Cancer.

[77] Kube D, Holtick U, Vockerodt M, Ahmadi T, Behrmann I, Heinrich PC (2001). STAT3 is constitutively activated in Hodgkin cell lines. Blood..

[78] Skinnider BF, Elia AJ, Gascoyne RD, Patterson B, Trümper L, Kapp U (2002). Signal transducer and activator of transcription 6 is frequently activated in Hodgkin and Reed-Sternberg cells of Hodgkin lymphoma. Blood..

[79] Dominguez-Sola D, Kung J, Holmes AB, Wells VA, Mo T, Basso K (2015). The FOXO1 transcription factor instructs the germinal center dark zone program. Immunity..

[80] Sander S, Chu VT, Yasuda T, Franklin A, Graf R, Calado DP (2015). PI3 kinase and FOXO1 transcription factor activity differentially control B cells in the germinal center light and dark zones. Immunity..

[81] Dutton A, Reynolds GM, Dawson CW, Young LS, Murray PG (2005). Constitutive activation of phosphatidyl-inositide 3 kinase contributes to the survival of Hodgkin’s lymphoma cells through a mechanism involving Akt kinase and mTOR. J Pathol.

[82] Xie L, Ushmorov A, Leithäuser F, Guan H, Steidl C, Farbinger J (2012). FOXO1 is a tumor suppressor in classical Hodgkin lymphoma. Blood..

[83] Vrzalikova K, Ibrahim M, Vockerodt M, Perry T, Margielewska S, Lupino L (2018). S1PR1 drives a feedforward signalling loop to regulate BATF3 and the transcriptional programme of Hodgkin lymphoma cells. Leukemia..

[84] Muppidi JR, Schmitz R, Green JA, Xiao W, Larsen AB, Braun SE (2014). Loss of signalling via Galpha13 in germinal centre B-cell-derived lymphoma. Nature..

[85] Renné C, Willenbrock K, Küppers R, Hansmann M-L, Bräuninger A (2005). Autocrine and paracrine activated receptor tyrosine kinases in classical Hodgkin lymphoma. Blood..

[86] Teofili L, Di Febo AL, Pierconti F, Maggiano N, Bendandi M, Rutella S (2001). Expression of the c-met proto-oncogene and its ligand, hepatocyte growth factor, in Hodgkin disease. Blood..

[87] Lamprecht B, Walter K, Kreher S, Kumar R, Hummel M, Lenze D (2010). Derepression of an endogenous long terminal repeat activates the CSF1R proto-oncogene in human lymphoma. Nat Med.

[88] Cader FZ, Vockerodt M, Bose S, Nagy E, Brundler MA, Kearns P (2013). The EBV oncogene LMP1 protects lymphoma cells from cell death through the collagen-mediated activation of DDR1. Blood..

[89] Moreau A, Praloran V, Berrada L, Coupey L, Gaillard F (1992). Immunohistochemical detection of cells positive for colony-stimulating factor 1 in lymph nodes from reactive lymphadenitis, and Hodgkin’s disease. Leukemia..

[90] Edginton-White B, Cauchy P, Assi SA, Hartmann S, Riggs AG, Mathas S (2019). Global long terminal repeat activation participates in establishing the unique gene expression programme of classical Hodgkin lymphoma. Leukemia..

[91] Renne C, Minner S, Küppers R, Hansmann ML, Bräuninger A (2008). Autocrine NGFbeta/TRKA signalling is an important survival factor for Hodgkin lymphoma derived cell lines. Leuk Res.

[92] Zheng B, Fiumara P, Li YV, Georgakis G, Snell V, Younes M (2003). MEK/ERK pathway is aberrantly active in Hodgkin disease: a signaling pathway shared by CD30, CD40, and RANK that regulates cell proliferation and survival. Blood..

[93] Juszczynski P, Ouyang J, Monti S, Rodig SJ, Takeyama K, Abramson J (2007). The AP1-dependent secretion of galectin-1 by Reed Sternberg cells fosters immune privilege in classical Hodgkin lymphoma. Proc Natl Acad Sci USA.

[94] Watanabe M, Ogawa Y, Ito K, Higashihara M, Kadin ME, Abraham LJ (2003). AP-1 mediated relief of repressive activity of the CD30 promoter microsatellite in Hodgkin and Reed-Sternberg cells. Am J Pathol.

[95] Jundt F, Anagnostopoulos I, Förster R, Mathas S, Stein H, Dörken B (2002). Activated Notch 1 signaling promotes tumor cell proliferation and survival in Hodgkin and anaplastic large cell lymphoma. Blood..

[96] Aldinucci D, Lorenzon D, Cattaruzza L, Pinto A, Gloghini A, Carbone A (2008). Expression of CCR5 receptors on Reed-Sternberg cells and Hodgkin lymphoma cell lines: involvement of CCL5/Rantes in tumor cell growth and microenvironmental interactions. Int J Cancer.

[97] Skinnider BF, Mak TW (2002). The role of cytokines in classical Hodgkin lymphoma. Blood..

[98] van den Berg A, Visser L, Poppema S (1999). High expression of the CC chemokine TARC in Reed-Sternberg cells. A possible explanation for the characteristic T-cell infiltration Hodgkin’s lymphoma. Am J Pathol.

[99] Hansen HP, Engels HM, Dams M, Paes Leme AF, Pauletti BA, Simhadri VL (2014). Protrusion-guided extracellular vesicles mediate CD30 trans-signalling in the microenvironment of Hodgkin’s lymphoma. J Pathol.

[100] Aoki T, Chong LC, Takata K, Milne K, Hav M, Colombo A (2020). Single-cell transcriptome analysis reveals disease-defining T-cell subsets in the tumor microenvironment of classic Hodgkin lymphoma. Cancer Discov.

[101] Wein F, Weniger MA, Hoing B, Arnolds J, Hüttmann A, Hansmann ML (2017). Complex immune evasion strategies in classical Hodgkin lymphoma. Cancer Immunol Res.

[102] Biggar RJ, Jaffe ES, Goedert JJ, Chaturvedi A, Pfeiffer R, Engels EA (2006). Hodgkin lymphoma and immunodeficiency in persons with HIV/AIDS. Blood..

[103] Wein F, Küppers R (2016). The role of T cells in the microenvironment of Hodgkin lymphoma. J Leukoc Biol.

[104] Ma Y, Visser L, Blokzijl T, Harms G, Atayar C, Poppema S (2008). The CD4+CD26- T-cell population in classical Hodgkin’s lymphoma displays a distinctive regulatory T-cell profile. Lab Invest.

[105] Roemer MGM, Redd RA, Cader FZ, Pak CJ, Abdelrahman S, Ouyang J (2018). Major histocompatibility complex class II and programmed death ligand 1 expression predict outcome after programmed death 1 blockade in classic Hodgkin lymphoma. J Clin Oncol.

[106] Bosshart H, Jarrett RF (1998). Deficient major histocompatibility complex class II antigen presentation in a subset of Hodgkin’s disease tumor cells. Blood..

[107] Greaves P, Clear A, Owen A, Iqbal S, Lee A, Matthews J (2013). Defining characteristics of classical Hodgkin lymphoma microenvironment T-helper cells. Blood..

[108] Poppema S. Immunobiology and pathophysiology of Hodgkin lymphomas. Hematology Am Soc Hematol Educ Program. 2005:231–8. 10.1182/asheducation-2005.1.231 16304386

[109] Cader FZ, Schackmann RCJ, Hu X, Wienand K, Redd R, Chapuy B (2018). Mass cytometry of Hodgkin lymphoma reveals a CD4(+) regulatory T-cell-rich and exhausted T-effector microenvironment. Blood..

[110] Lee SP, Constandinou CM, Thomas WA, Croom-Carter D, Blake NW, Murray PG (1998). Antigen presenting phenotype of Hodgkin Reed-Sternberg cells: analysis of the HLA class I processing pathway and the effects of interleukin-10 on epstein-barr virus-specific cytotoxic T-cell recognition. Blood..

[111] Oudejans JJ, Jiwa NM, Kummer JA, Horstman A, Vos W, Baak JP (1996). Analysis of major histocompatibility complex class I expression on Reed-Sternberg cells in relation to the cytotoxic T-cell response in Epstein-Barr virus-positive and -negative Hodgkin’s disease. Blood..

[112] Gandhi MK, Moll G, Smith C, Dua U, Lambley E, Ramuz O (2007). Galectin-1 mediated suppression of Epstein-Barr virus specific T-cell immunity in classic Hodgkin lymphoma. Blood..

[113] Yamamoto R, Nishikori M, Kitawaki T, Sakai T, Hishizawa M, Tashima M (2008). PD-1-PD-1 ligand interaction contributes to immunosuppressive microenvironment of Hodgkin lymphoma. Blood..

[114] Vari F, Arpon D, Keane C, Hertzberg MS, Talaulikar D, Jain S (2018). Immune evasion via PD-1/PD-L1 on NK cells and monocyte/macrophages is more prominent in Hodgkin lymphoma than DLBCL. Blood..

[115] Kawashima M, Carreras J, Higuchi H, Kotaki R, Hoshina T, Okuyama K (2020). PD-L1/L2 protein levels rapidly increase on monocytes via trogocytosis from tumor cells in classical Hodgkin lymphoma. Leukemia..

[116] Ho WT, Pang WL, Chong SM, Castella A, Al-Salam S, Tan TE (2013). Expression of CD137 on Hodgkin and Reed-Sternberg cells inhibits T-cell activation by eliminating CD137 ligand expression. Cancer Res.

[117] Ishida T, Ishii T, Inagaki A, Yano H, Komatsu H, Iida S (2006). Specific recruitment of CC chemokine receptor 4-positive regulatory T cells in Hodgkin lymphoma fosters immune privilege. Cancer Res.

[118] Marshall NA, Christie LE, Munro LR, Culligan DJ, Johnston PW, Barker RN (2004). Immunosuppressive regulatory T cells are abundant in the reactive lymphocytes of Hodgkin lymphoma. Blood..

[119] Gandhi MK, Lambley E, Duraiswamy J, Dua U, Smith C, Elliott S (2006). Expression of LAG-3 by tumor-infiltrating lymphocytes is coincident with the suppression of latent membrane antigen-specific CD8+ T-cell function in Hodgkin lymphoma patients. Blood..

[120] Patel SS, Weirather JL, Lipschitz M, Lako A, Chen PH, Griffin GK (2019). The microenvironmental niche in classic Hodgkin lymphoma is enriched for CTLA-4-positive T cells that are PD-1-negative. Blood..

[121] Choe JY, Yun JY, Jeon YK, Kim SH, Park G, Huh JR (2014). Indoleamine 2,3-dioxygenase (IDO) is frequently expressed in stromal cells of Hodgkin lymphoma and is associated with adverse clinical features: a retrospective cohort study. BMC Cancer.

[122] Reinke S, Brockelmann PJ, Iaccarino I, Garcia-Marquez MA, Borchmann S, Jochims F, et al. Tumor and microenvironment response but no cytotoxic T-cell activation in classic Hodgkin lymphoma treated with anti-PD1. Blood. 2020;136:2851–63. 10.1182/blood.2020008553 33113552

[123] Nagasaki J, Togashi Y, Sugawara T, Itami M, Yamauchi N, Yuda J (2020). The critical role of CD4+ T cells in PD-1 blockade against MHC-II-expressing tumors such as classic Hodgkin lymphoma. Blood Adv.

[124] Cader FZ, Hu X, Goh WL, Wienand K, Ouyang J, Mandato E (2020). A peripheral immune signature of responsiveness to PD-1 blockade in patients with classical Hodgkin lymphoma. Nat Med.

[125] Jalali S, Price-Troska T, Bothun C, Villasboas J, Kim HJ, Yang ZZ (2019). Reverse signaling via PD-L1 supports malignant cell growth and survival in classical Hodgkin lymphoma. Blood. Cancer J.

[126] Du J, Neuenschwander M, Yu Y, Dabritz JH, Neuendorff NR, Schleich K (2017). Pharmacological restoration and therapeutic targeting of the B-cell phenotype in classical Hodgkin lymphoma. Blood..

[127] Guan H, Xie L, Wirth T, Ushmorov A (2016). Repression of TCF3/E2A contributes to Hodgkin lymphomagenesis. Oncotarget..

[128] Yuki H, Ueno S, Tatetsu H, Niiro H, Iino T, Endo S (2013). PU.1 is a potent tumor suppressor in classical Hodgkin lymphoma cells. Blood..

[129] Küppers R (2005). Mechanisms of B-cell lymphoma pathogenesis. Nat Rev Cancer.

[130] Spina V, Bruscaggin A, Cuccaro A, Martini M, Di Trani M, Forestieri G (2018). Circulating tumor DNA reveals genetics, clonal evolution, and residual disease in classical Hodgkin lymphoma. Blood..

